# Social goals under a neoliberal agenda: measures to promote equality in European higher education read through a Foucauldian lens

**DOI:** 10.3389/fsoc.2025.1492863

**Published:** 2025-02-10

**Authors:** Elena-Loreni Baciu, Theofild-Andrei Lazăr, Raluca Iunia Totan

**Affiliations:** The Research-Action Centre on Discrimination and Social Inclusion, Department of Social Work, Faculty of Sociology and Psychology, West University of Timișoara, Timișoara, Romania

**Keywords:** European higher education, vulnerable students, governmentality, Foucault, European higher education policies, social dimension of higher education, neoliberal policies

## Abstract

In this study we draw on Foucault’s work on governmentality and examine the power dynamics involved in establishing and implementing policies that promote equality in European higher education. Using a qualitative case study design, we selected 17 public universities situated in 13 European countries, from which we collected information about (1) the way these institutions problematize inequality in reference to participation in higher education, by labeling and categorizing vulnerable students and (2) the modes of governing and power tools (designed as support measures) they employ to address inequality. The results of the study show that the most typical profiles of vulnerability with which the universities in the sample engage include: students with disabilities, students from low-income backgrounds and students with children. Additionally, most universities use targeted support measures (as opposed to mainstreaming strategies) which consist in a mix of financial aid and support and adaptation services. The critical analysis of these measures reveals their power to shape students’ identifies and actions, through processes of subjectification, categorization, normalization and responsabilization. In the last section, we discuss the tension that appears between the European universities’ social dimension and the neoliberal policies that shape their functioning.

## 1 Introduction

### 1.1 The need for a more equitable access to higher education

In an academic setting, as per the guidelines set forth by UNESCO, through the 4th UN Sustainable Development Goal, the promotion of diversity relates to the equitable facilitation of opportunities, engagement, advancement, and academic achievement for students from vulnerable groups. Brennan and Naidoo (2008) note that the recent expansion of enrolments in higher education does not necessarily translate into a higher level of social equity, meaning that the access of vulnerable groups does not increase with it. Under this framework, the objective is to strive for a student body that accurately mirrors the proportional representation of minority groups within society (United Nations Educational, Scientific and Cultural Organization, 2009). The attainment of this objective needs a proactive engagement of universities in the implementation of measures and policies aimed at fostering inclusivity and providing support to vulnerable student populations. Scholars agree on the benefits of pursuing this aim, by arguing its positive impact at individual, as well as at institutional and societal levels. Individual advantages pertain to observing the positive effects of pursuing higher education, which encompass enhanced health outcomes, increased earning capacity, as well as higher levels of life satisfaction (Salmi, 2018a; Salmi and D'Addio, 2021), and well-being (Tonon, 2021). Moreover, research suggests that students who engage in frequent interactions with peers from diverse economic backgrounds and cultures tend to exhibit greater levels of tolerance, improved self-esteem and academic performance, enhanced teamwork skills, and a heightened commitment to social responsibility (Hurtado and Deangelo, 2012). Other potential advantage is that a broader range of experiences, abilities, and thinking styles can enhance individuals’ capacity to comprehend and resolve problems (Gorad and Smith, 2006). The increase of the person’s educational attainment is associated with several financial positive outcomes, including diminished rates of unemployment, heightened tax revenues, enhanced intergenerational mobility, increased engagement in civic activities, and decreased reliance on social assistance programs (Salmi, 2018a; Salmi and D'Addio, 2021). These factors will, in turn, have an impact on the wider social climate. The labor market will experience an increase in the quantity of skilled and competent individuals, leading to a notable potential for innovation. Consequently, the overall standard of living for all members of society is expected to rise (Bowman, 2011). Therefore, given the wide-ranging individual and societal advantages that arise from it, it becomes imperative to ensure equitable access to higher education, in striving to attain social equality and equity. Education should be regarded as a public good, universally accessible, as it cultivates responsible people who uphold and reinforce democracy (Toliou, 2007).

In reference to higher education, a person is considered to belong to a vulnerable group when a number of variables (economic, social, medical, etc.) limit that person’s possibility of pursuing higher education, thus resulting in a lower level of participation in higher education of members of that specific group (compared to members of other groups). In this discussion framework about access to higher education, the term “vulnerable groups” is often used inter-changeably with “underrepresented” or “socially excluded” groups. Currently, there is no shared definition or list of the vulnerable in higher education (Salmi and D'Addio, 2021). Instead, each country establishes its own criteria for identifying these groups, taking into account the unique social context within which they operate. Typically, authors identify the following groups as under-represented in higher education and in need of support (Salmi and D'Addio, 2021): people from low-income households; women; people from vulnerable minority groups (from an ethnic, linguistic, religious, cultural, or place-of-residence perspective); people with disabilities. Some of these characteristics co-occur in the same individual, creating an intersection of vulnerabilities that impedes that individual’s access to higher education (e.g., female, disabled, and low-income students).

Several studies (Vlk and Stiburek, 2018; Marginson, 2016; Salmi, 2018b) point out that national governments and universities are implementing a variety of strategies to increase the participation of students from vulnerable populations in higher education. These solutions include financial measures (incentives for both universities and students, ranging from various funding formulas to funding projects or tuition fees, or awarding scholarships), information and support measures (mentoring, counseling, consulting), and organizational changes (such as increasing the flexibility of learning paths, changes in curricula, revising admission criteria, and implementing quality assurance procedures). Based on the results of a survey conducted in 71 countries, Salmi (2018b) classifies the measures used by governments and higher education institutions to increase opportunities for disadvantaged students in two basic categories: monetary measures (such as grants/scholarships and student loans) and non-monetary programs (like affirmative action, reformed admission criteria, outreach and bridge programs, or retention programs) (Salmi and D'Addio, 2021).

Additionally, when enrolled in higher education, students from vulnerable groups have generally lower completion rates compared to those of students from non-vulnerable groups (Salmi, 2020). Thus, governments and universities provide support measures meant to assist students from vulnerable categories who have accessed higher education to complete their study program.

### 1.2 The usefulness of a Foucauldian lens in examining educational policies

Michel Foucault’s research on the modern history of exclusion is essential to his methodology for comprehending the evolution of modern institutions in emergent liberal societies. Foucault highlights that while individuals may have a clear understanding of their acts at a small scale (local level), there is a lack of coordination of the wider consequences of these actions (Nicoll and Fejes, 2008). From a Foucauldian perspective, higher education is an integral part of modern political technologies and power strategies (Nicoll and Fejes, 2008). He considers that political power is not exercised exclusively through institutions directly subordinated to the government (such as public administration, the police or the military), but is also exercised through the mediation of institutions apparently independent from the political class, which justify their purpose as the dissemination of knowledge, while in fact, advancing a political agenda designed to maintain the status-quo of the power hierarchy and to exclude the power instruments of other social classes. He specifically mentions that educational institutions are the best examples of this category.

From this stance, the study of policy through a Foucauldian lens goes beyond the narrow focus on government institutions and aims to examine the various social knowledge bases that inform government policy decisions. Nicoll and Fejes (2008) highlight the usefulness of a post-structural Foucauldian perspective in critically analyzing the validity of pre-established notions accepted as general truths in education policies.

From Foucault’s perspective power operates through disciplinary mechanisms. He introduces the concept of the *“panopticon,”* a metaphor for how power is exercised through surveillance, leading to self-regulation and the creation of *“docile bodies”* (Foucault, 1995). Power is not inherent to an individual or entity. Instead, power is based on relationships and communication. It permeates all areas, traversing interconnected networks and functioning through systems of authority. From a Foucauldian perspective, the intricate dynamics of power and the various systems of control and management in education contribute to normalization of behaviours and identities (Hannus and Simola, 2010).

The concept of *normalization* refers to the process of setting standards of what is considered “normal” and “abnormal,” with the aim of creating the “docile bodies” that conform to societal expectations (Foucault, 1995). This concept provides us with a means to analyze the categorization of various groups of students, the criteria used to label certain categories as “vulnerable”, and the logic of the distribution of resources among groups, designed to (re) shape equality (Hannus and Simola, 2010).

In his work, Foucault also introduced the concept of “governmentality” to discuss the comprehensive nature of governance, as an authoritative attempt to regulate the behavior. The term “*governmentality*” (Foucault, 1991, 2000) refers to institutions’ endeavours to shape individual behavior through various forms of regulation and intervention (Dean, 2009). A *governmentality analysis* focuses on how individuals are subjected to various processes and become objects of knowledge within specific historical contexts (Nicoll and Fejes, 2008).

The term *“mode of governing”* encompasses not only the mental aspects but also the technologies, techniques, and various dimensions of power, such as those related to the subject, ethics, and visibility (Foucault, 1997). The focus is both on the complex and layered structure of a governing system, comprising various forms and dimensions of power (Hannus and Simola, 2010), and also on the ways rule is justified, specifically focusing on governmental rationalities or governmentalities (Bacchi, 2010).

According to Foucault, discipline and regulation refer to the methods by which power is exercised to take care of various aspects of life. They encompass the entire area between the organic and the biological, between individual bodies and populations. From his perspective, *biopolitics* is a form of managing life—optimizing health, regulating birth rates, controlling mortality, and ensuring the well-being of the population, targeting the population as a whole, rather than individuals alone (Foucault et al., 2008). Foucault (2003) argues that *bio-power* arises inside the modern state as a well-organized political technique rooted in disciplinary power. It involves a focus on the human species and a desire to control and exploit the body. The concept of bio-power led to the development of the perspective on education as a collaborative initiative including both the government and individuals. The population was to be controlled by means of education, in which authority was exerted on individuals’ bodies (discipline) in order to mold them in specified ways. In his later work (Foucault et al., 2008), the relationship between biopolitics and neoliberalism is explored, analyzing how economic policies and government practices intertwine with biopolitical strategies to regulate and control populations.

Foucault also employs the term *discourse* in his work. From his perspective, discourse refers to the framework in which certain assertions are regarded as true, and it does not exist within itself, but rather allows the object to become visible or manifest: discourse generates the entities that it discusses, as well as the topics (Ball, 2019). Carol Bacchi, a post-structuralist scholar heavily influenced by Foucault, has developed the *policy-as-discourse* framework. This framework highlights the role of language and discourse in shaping the boundaries of what can be expressed, thought, and enacted in policy discussions (Bacchi, 2000). Policy plays a crucial role in defining and shaping both solutions and problems. In other words, policy serves not to address already existing real-world issues, but to establish both the issues and their potential resolutions (Holloway et al., 2024).

### 1.3 The social dimension of European higher education, under neoliberal policies

The present operational landscape of European universities is significantly influenced by the rapid transformations occurring in their societal milieu. These changes encompass various aspects, including the social ramifications of heightened social inequality, the decline in the middle-class population alongside the rise in individuals at risk of poverty, demographic aging and population decrease, shifts in population lifestyles, the refugee crises, the escalation of nationalist and xenophobic sentiments (Curaj et al., 2018). Therefore, the role of universities in society, much like society itself, is continuously transforming, diversifying, and adapting.

From an historical perspective, the preoccupation of the European states with enhancing the access of vulnerable groups to higher education, stemmed from the diffusion of the social exclusion paradigm in the European space. This began in the mid-1980s, when the need of incorporating a robust “social dimension” into the European project was increasingly stressed in the political discourse. From this standpoint, the narrative of the need to *fight against social exclusion* progressively evolved into a European policy model which further shaped social and political action (Béland, 2007).

Various authors (Pasias and Roussakis, 2012; Toliou, 2007; Addison, 2002) point out that, in the last recent decades, the European educational policies have explicitly adopted a pronounced neoliberal economic model, and have willingly submitted “science, research and knowledge” (Toliou, 2007: p. 51) to the macro-economic agenda by gradually transforming into proactive employment strategies (Lavdas et al., 2007; Papadakis and Drakaki, 2023). Neoliberal modes of governance are becoming increasingly visible in the educational sector of the European Commission. The policies and programs related to education and training are becoming focused almost exclusively on cultivating mobile, adaptable, and self-directed European workers, rather than emphasizing institutionalized personal development (Addison, 2002). Although the Europe2020 Strategy has enhanced its social dimension relative to the Lisbon Strategy, by highlighting, at least to some extent, social inclusion, the prevalence of economic rationality persists (Papadakis and Drakaki, 2023).

Biesta (2015) posits that developments in education are situated within a broader societal framework, which shifted from welfarism to new managerialism. This shift has replaced the public service ethos characterized by equity, care, and social justice with an ethos focused on efficiency and free market competition. Papadakis and Drakaki (2023) note that all the three encompassing dimensions of neoliberalism (ideology, policy framework, and model of governmentality) have influenced the effort for a unified European Strategy in Education and Training. The dominance of economic reasoning within the public policy framework exacerbated the underlying institutional and regulatory imbalances between the Member States, while education policy became increasingly constrained by a singular macroeconomic agenda.

One of the current ambitions declared by the European Higher Education Area is to expand the *social dimension of higher education*, which translates into policies which accentuate the need for European universities to become increasingly engaged with their social mission of building an *inclusive society* based on democratic values and respect for human rights. The social dimension encompasses a significant aspect that pertains to the reduction of disparities in the attainment of higher education. This aspect is supported by two key arguments, namely justice and efficiency, as outlined by Salmi (2018a). This attribute is regarded as a primary differentiating factor that sets apart the European approach to the role of higher education from other regions, such as the United States, where the prevailing perspective on the functioning of tertiary education sector tends to be, under the influence of neoliberal doctrines, predominantly commercial in nature (Scott, 2022).

Several scholars who have examined the impact of the Bologna process and the European Higher Education Area on the social dimension of European universities have reached the consensus that the development of the social dimension in higher education is the most challenging aspect of the Bologna process (Vlk and Stiburek, 2018), since so far, its effects are primarily symbolic rather than tangible. This is proven by the lack of substantial concrete outcomes in this regard and the existence of enduring disparities between policies and practices (Vlk and Stiburek, 2018; Curaj et al., 2018; Scott, 2022). In this context, we consider that a close examination of the relationships between policies and practices regarding the enactment of the social dimension by European universities it’s needed.

One important question that scholars in the field of education policy have raised is how policies and policy conditions have the potential to fundamentally alter not just the actions of educational actors, but also their identities. This line of inquiry has been explored by post-structuralist scholars, including Ball (2003) and Holloway and Brass (2018). These types of questions are valuable for examining the underlying principles of policies, rather than focusing on specific outcomes or effects. This has significant implications for considering how policy discourses are transferred and circulated across various contexts. When considering these questions, the researcher’s focus is not on the effectiveness of a specific policy or whether it requires modification or elimination. Their concerns revolve around the process by which certain logics and rationalities gradually gain acceptance as the norm, ultimately influencing what is deemed a necessary solution, often in the form of a policy (Holloway et al., 2024).

Scholars who sought to critically analyze the effects of educational policies in terms of promoting equality and social inclusion have extensively drawn from Michel Foucault’s work and especially on two aspects: his analysis of the interplay between power and discipline, and his exploration of the concept of governmentality (Ball, 2019).

Gravaris (2005) notes that the integration of educational policy with active employment policy has enabled the macroeconomic “surveillance” of public policy in education. Higher education institutions, together with their staff and students, are consistently urged to assume accountability for their financial prosperity and social viability (Hellbert-Steurer, 2018). The neoliberal agenda fosters fragmented and utilitarian knowledge while also preparing students for certain roles. Education increasingly functions merely as a mechanism for generating skilled professionals for the labor market (Toliou, 2007). Neoliberal strategies for managing human capital and promoting self-entrepreneurialism encompass auditing, accounting, and quality assessment. These strategies are extensively employed to render individuals measurable, quantifiable, and accountable, hence promoting self-regulation and entrepreneurialism. The quantification and assessment of indicators to evaluate the attainment of EU objectives, along with the monitoring of EU guideline implementation, endorse neoliberal auditing and accounting practices (Hellbert-Steurer, 2018).

Power has moved from human and social science knowledge, which was previously dominant under the welfare state, to numeric regimes that facilitate control through normative numbers. The subjectifying power of these numeric regimes stems from their assertions of neutrality, and objectivity (Hellbert-Steurer, 2018; Rose, 1996).

Robertson and Dale (2002) observe that the technologies of power employed by the neoliberal state serve as discursive and strategic instruments used to address various educational issues and conflicts. The de-politization of education is conducted through discursive and policy strategies that highlight self-responsibility and self-regulation: educational institutions are required to function as entrepreneurs in pursuit of their own interests to enhance competitiveness and efficiency. Neoliberal rationality asserts that this behavior aligns with the general will of society, obscuring the realities of exclusion, educational failure, and segregation resulting from educational markets (Bonal, 2003).

Through this study, we employ a Foucauldian lens in examining the rationales and modes of governing behind the tools through which public European universities put their social dimension into practice, in relation to the policies aimed to facilitate access in higher education for students from vulnerable categories. We are interested in exploring the broader implications of establishing and implementing policies to promote equality in higher education, as these strategies are deeply intertwined with and essential to power dynamics in our current society.

The paper is organized into 4 distinct sections. In Section 2, and following the Introduction, we describe the methodological and analytical approach of the research. Section 3 presents the findings of the study. In Section 4 we conduct a critical analysis of the results in the reference to the Foucauldian approach on power dynamics and discuss the wider implications of the rationales and modes of governing employed by European Universities in reference to the students from vulnerable groups.

## 2 Materials and methods

### 2.1 Methodology of the study

The purpose of this study was to analyze the ways in which public European universities problematize inequality in higher education and the modes of governing they employ to address it. Through it, we seek to prompt reflection on the justification and impact of regulations and practices on students’ identities and agency. Bacchi (2010) proposes that, to gain a better understanding of the framework in which rule occurs, it is advisable to thoroughly examine and question problematizations. This approach emphasizes the importance of relations of power, which are often overlooked in the everyday implementation of inclusive policies and practices, as well as in research on the topic. Instead of evaluating the effectiveness or ineffectiveness of measures aimed at supporting access and success in higher education for vulnerable groups, or categorizing it as inherently good or bad, we propose different questions:

*RQ1.* How do European universities problematize inequality in reference to access and success to higher education, by labeling and categorizing vulnerable students?

*RQ2.* What modes of governing (designed as support measures) do European universities employ in addressing inequality, in reference to access and success to higher education?

In this inquiry we used a qualitative research methodology that pertains to the comparative case studies (Knight, 2001). It entails the examination of two or more cases that possess some shared characteristics while also exhibiting distinct differences. The comparative case study methodology concurrently addresses macro, meso, and micro dimensions of case-based research and employs two comparative logics (Bartlett and Vavrus, 2017): firstly, the conventional “compare and contrast” framework; and secondly, a “tracing across” approach using various places or scales. The objective of this approach is to analyze the similarities and differences among cases, as well as to understand the reasons and effects of these differences. We treated each university included in the sample as a separate case study.

### 2.2 Selection criteria for sampled universities

In discussing the ways to overcome the many practical restrictions that researchers encounter when doing case-study research, Koivu and Hinze (2017) recommend placing similar importance on logistical factors as on methodological concerns, and urge researchers to enhance transparency regarding study objectives and the selection of cases. In line with these recommendations, we established the following selection criteria for the universities included in the analysis:

Criteria with methodological relevance:

1. be public universities (as opposed to private)—due to the fact they are part of the public sector, their policies are drawn from and compatible with wider political technologies and power strategies, thus better reflecting the overall political ideology.
2. be varied from each other in terms of number of students—Flyvbjerg (2006) recommends the use of a maximum variation case selection method, which involves the deliberate selection of examples that encompass a wide range of units of analysis.
3. be sufficiently spread geographically around Europe, as to cover various types of welfare regime arrangements (Esping-Andersen, 1990; Rostila, 2007) and government agendas—although fairly homogeneous in some aspects, the European universities are also, at the same time, very different from each other, in other aspects, due to factors such as primary function, hierarchy structure, extent of state control, social and economic roles, local cultures and practices etc. (Brennan and Naidoo, 2008; Hüther and Krücken, 2016; Willemse and De Beer, 2012).

Criteria with logistical relevance:

1. have enough information posted on their official website as to allow the data collection process—Due to principles of transparency, accessibility and public accountability, universities are increasingly presenting themselves to the public through their websites. Although there are still some needed improvements in enhancing accessibility of university websites (Campoverde-Molina et al., 2023), official websites of public entities are a credible source of information about the institutions’ activities, processes and structures and there is a growing trend among researchers to use them as a reliable source of data (Nash and Churchill, 2020; Cuan-Baltazar et al., 2020; Peeri et al., 2020).
2. have a version of their official website in a language that was familiar to the research team (English, French, Italian or Romanian)—the internationalization ambitions of universities (van den Hende et al., 2023; Bulut-Sahin and Kondakci, 2022) have resulted in increased multi-lingual versions of their official websites. Also, English, French, and Italian are among the languages most used Europe (Eurostat, 2022).

We started to build the sample based on the structure of an existing European Alliance, constituted under the European Universities initiative (European Commission, 2020), from 6 universities in Europe, that fit the selection criteria, and expanded it from there.

After applying these criteria, a sample of 17 public universities from 13 European countries resulted. Their list and the main characteristics that led to their inclusion in the sample are shown in Table 1.

**Table 1:** Characteristics of the universities included in the sample.

Name code	Approx. no. of students	Country	Type of welfare regime
University 1	30.000	Sweden	Social-democratic
University 2	11.000	Norway	
University 3	35.000	Finland	
University 4	16.000	United Kingdom of Great Britain and Northern Ireland	Liberal
University 5	22.000	United Kingdom of Great Britain and Northern Ireland	
University 6	38.000	Republic of Ireland	
University 7	14.000	France	Conservative
University 8	15.000	France	
University 9	50.000	Germany	
University 10	7.000	Portugal	Mediterranean
University 11	70.000	Italy	
University 12	90.000	Italy	
University 13	36.000	Spain	
University 14	23.000	Hungary	Post-socialist
University 15	23.000	Slovakia	
University 16	32.000	Romania	
University 17	15.000	Romania	

Based on the approx. Number of students, the universities’ sizes varied between small (7.000 students) and very large (90.000 students) and were distributed as follows: six universities (located in Norway, UK, France, Portugal, and Romania) had less than 20.000 students; eight universities (located in Sweden, Finland, UK, Ireland, Spain, Hungary, Slovakia and Romania) had between 20.000 and 40.000 students, and three universities (located in Italy and Germany) had more than 40.000 students.

Based on the geographical positioning and association with a certain welfare regime type of the countries they belonged to, the distribution of the universities in the sample was the following: three universities situated in three countries from Northern Europe (Sweden, Norway and Finland, associated with the social-democratic welfare regime), three universities situated in UK and the Republic of Ireland (associated with the liberal welfare regime), three universities situated in France and Germany (associated with the continental or conservative regime), four universities situated in three countries from Southern Europe (Portugal, Italy and Spain, associated with the Mediterranean regime) and four universities situated in three countries from Central and Eastern Europe (Hungary, Slovakia, and Romania, associated with the post-socialist regime).

### 2.3 Data collection and analysis

At each of these universities, we identified and analyzed the support measures provided to students from vulnerable categories. We considered these measures as operational tools of governance, that are reflective of wider policies. The identification of the support measures was carried out by consulting the documents and information posted on the official websites of the institutions. We classified the support measures in two initial categories: (1) financial aid and (2) support and adaptation services.

**Figure 1:**
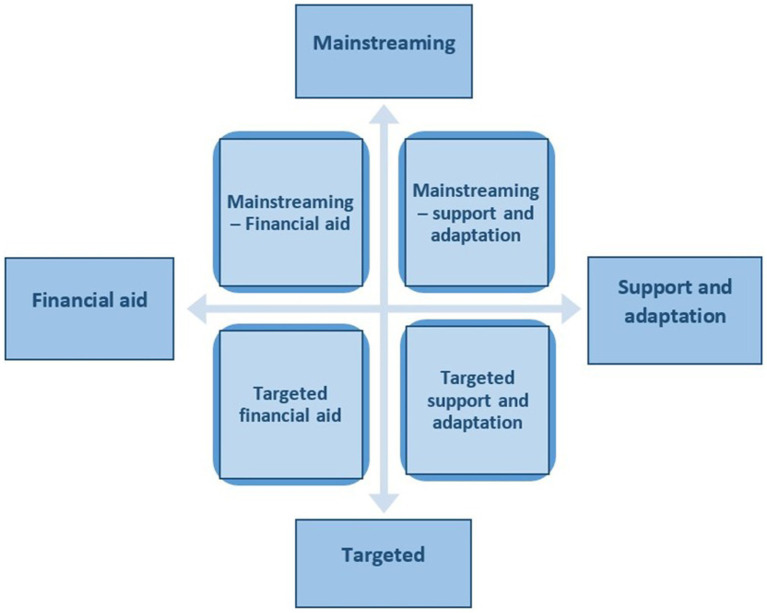
The two main axes of analysis for the data collected.

For the analysis of the data, we used the inductive exploration of the qualitative material and categorical aggregation (Stake, 1995), seeking to collect and group among the cases those instances that are relevant for the two main research questions. Later on, in the comparative analysis of the two categories of measures, after the authors familiarized themselves with the content of the data collected, we observed that some measures were dedicated to all students (without specific pre-established eligibility criteria), while others targeted very specific categories (such as students with disabilities). In order to take this structural aspect into account, we further sub-divided the identified measures in two categories: mainstreaming vs. targeted. Thus, in the end, the data was placed in a 2×2 matrix, resulted from the intersection of the two main axes of analysis (type of measure—financial vs. support and adaptation; and beneficiary of the measure—mainstream vs. targeted) (see Figure 1).

Various authors (Yin, 2009; Creswell, 2013) suggest that this cross-case synthesis is a useful analytic technique when the inquiry involves two or more cases, because it allows the research to identify more easily the similarities and differences among the cases. Thus, four analytical categories resulted: (1) Mainstreaming financial aid measures; (2) Targeted financial aid measures; (3) Mainstreaming support and adaptation services; and (4) Targeted support and adaptation services. The synthesis of the results for each category is presented in Table 2.

**Table 2:** Distribution of support measures by type and recipients.

Name code	Country	Type of welfare regime	Mainstreaming-financial aid	Targeted—financial aid	Mainstreaming-support and adaptation	Targeted—support and adaptation
University 1	Sweden	Social-democratic	No	No	Ombudsman	Students with special needs (adapted learning materials, assistive software, mentorship, adapted examination, infrastructure adaptation, including in student dorms)
University 2	Norway		Preferential rates on accommodation and canteen	No	Ombudsman	Students with special needs (adapted learning materials, assistive software, adapted examination, infrastructure adaptation, including in student dorms) Students with children (priority at accommodation in student dorms, kindergarten services)
University 3	Finland		Scholarships Preferential rates on accommodation and canteen	No	Ombudsman Support and counseling for equal treatment	Students with special needs (adapted learning materials, adapted examination)
University 4	United Kingdom of Great Britain and Northern Ireland	Liberal	No	Students with special needs (scholarships). Students from vulnerable families or disadvantaged communities (scholarships). Students with children (scholarships).	Counseling and welfare services Induction and foundational programs Labor market integration assistance Peer assisted learning	Students with special needs (adapted accommodation, adapted learning materials, assistive software, adapted examination) Students from vulnerable families or disadvantaged communities (program to prepare pupils 12 to 18 to access higher education)
University 5	United Kingdom of Great Britain and Northern Ireland		Preferential rates on loans Preferential rates on accommodation	Students with special needs (grants and scholarships) Students with children and other dependents (grants and scholarships)	Equivalent of Ombudsman Mental health support services Guidance in formulating complaints	Students with special needs (adapted learning materials, assistive software, mentorship, adapted examination, infrastructure adaptation, including in student dorms)
University 6	Republic of Ireland		Support funds for unforeseen events Support funds for part-time students	Students with children (support funds)	Equivalent of Ombudsman Emergency help-line Mental health support services Tutoring Counseling Assistance for victims of harassment and abuse Spiritual services Guidance in formulating complaints	Students from ethnic minorities (working groups and training modules mainly for staff, to combat discrimination)
University 7	France	Conservative	No	Pregnant students or students with children (reduced fees) Students with special needs (scholarships) Students from low-income families (scholarships)	No	Students with children (partial or total exemption of obligations to attend; adapted examination sessions) Students with special needs (adapted learning materials, assistive software, counseling, adapted examination, infrastructure adaptation, including in student dorms; partial or total exemption of obligations to attend; academic and career guidance; arrangements for transport)
University 8	France		No	Students with disabilities (allowances, subsidies, indemnities)	No	Students with disabilities (adapted learning materials, assistive software, counseling, adapted examination, assistance—including medical, infrastructure adaptation, including in student dorms; career guidance)
University 9	Germany		No	Students with disabilities (state scholarships) Students with children (care fund) Students from vulnerable families—first generation students (employment opportunities within university structures)	Ombudsman	Students with special needs (adapted learning materials, partial exemption of obligations to attend; assistive software, counseling, adapted examination, assistance, infrastructure adaptation, including in student dorms; career guidance) Students with children (Maternity leave for pregnant students; Provision of nursery services, including emergency child care; Designated spaces in the university buildings for breastfeeding or changing the child’s diapers)
University 10	Portugal	Mediterranean	No	Students with special needs (reserved seats upon admission) Students from low-income families (social support funds; solidarity funds; scholarships) Students from rural areas (scholarships)	Ombudsman	Students with special needs (counseling; priority in accommodation; adaptations of teaching and examination process; pedagogical support)
University 11	Italy		No	Students with special needs (exemption from some fees)	No	Students with special needs (adapted learning materials; Tutorship; arrangements for transport; adaptation software; adapted examination; assistance, including for personal hygiene; infrastructure adaptation; career guidance)
University 12	Italy		Paid tutorial activities Grants, prizes and scholarships Discounted transportation Preferential rates at canteens	Refugee students (grants) Students with children (reduced school fees) Students with special needs (exemptions from school fees; financial support to purchase technical solutions to adapt study) Students from vulnerable (low-income) families (exemption from school fees, in case they comply with academic performance criteria)	Ombudsman Mediation for employment Career guidance Psychological support Medical assistance Designated spaces in the university buildings for breastfeeding or changing the child’s diapers	Students with special needs (adapted admission; adapted examination; adapted attendance requirements; assistance in selecting and using assistive technologies; infrastructure adaptation; career guidance; special arrangements regarding the duration of studies) Students from vulnerable (low-income) families Employment opportunities within university structures (if certain academic performance criteria are met) Students with children or other dependents (Counseling and psychological support services)
University 13	Spain		No	Students with special needs (scholarships, reserved seats) Students from vulnerable (low-income) families (scholarships) Students from rural areas (scholarships)	Psychological guidance and counseling	Students with special needs (adapted examination; personal and technical support for autonomy in the classroom)
University 14	Hungary	Post-socialist	Community scholarships (based on various performance criteria) Scholarships based on criteria related to nationality, citizenship or religion Performance scholarships	Students with vulnerable social backgrounds (scholarships)	Counseling services	No
University 15	Slovakia		No	No	Gender equality promotion program Psychological counseling	Students with special needs (adapted accommodation for students and their caretakers; Provision of assistive technology, including advice on its use; tutoring and meditation services; Academic and professional counseling and guidance; Adaptation of the learning process; Pedagogical support; Mediation with teaching staff)
University 16	Romania		Scholarships for study and performance Scholarships for occasional support (in special circumstances) Scholarships for student projects Free of charge camps Medical assistance Reduced rates or fee exemptions on public transport Accommodation in student campus	Students who are orphaned or are beneficiaries of the child protection system (reduced or exemption from study fees; reduced rates or free accommodation; scholarships; reserved seats) Students from rural areas and ethnic minority students (reserved seats) Students with special needs (reduced rates or free accommodation; scholarships) Students with children (reserved accommodation quota) Students with chronic illnesses (reserved accommodation quota; scholarships) Students from low-income families (reduced rates or free accommodation; scholarships)	Tutoring programs Academic and career guidance service	No
University 17	Romania		Scholarships for study and performance Free of charge camps Medical assistance Reduced rates or fee exemptions on public transport Accommodation in student campus	Students with special needs or chronic illnesses (reduced rates or free accommodation; scholarships; exemptions from or reduced study fees) Students who are orphaned or are beneficiaries of the child protection system (reduced rates or free accommodation; scholarships; reserved seats) Students from low-income families (reduced rates or free accommodation; scholarships) Students from Roma ethnic minority (reserved seats) Students from rural areas (reserved seats)	Academic and career guidance service Support for dropout prevention Support for future students (currently pupils) to access higher education	Students with special needs (counseling services; support for adaptation to academic requests; accessibilization of infrastructure; assistive software)

## 3 Results

### 3.1 Categorization of vulnerable students

**Figure 2:**
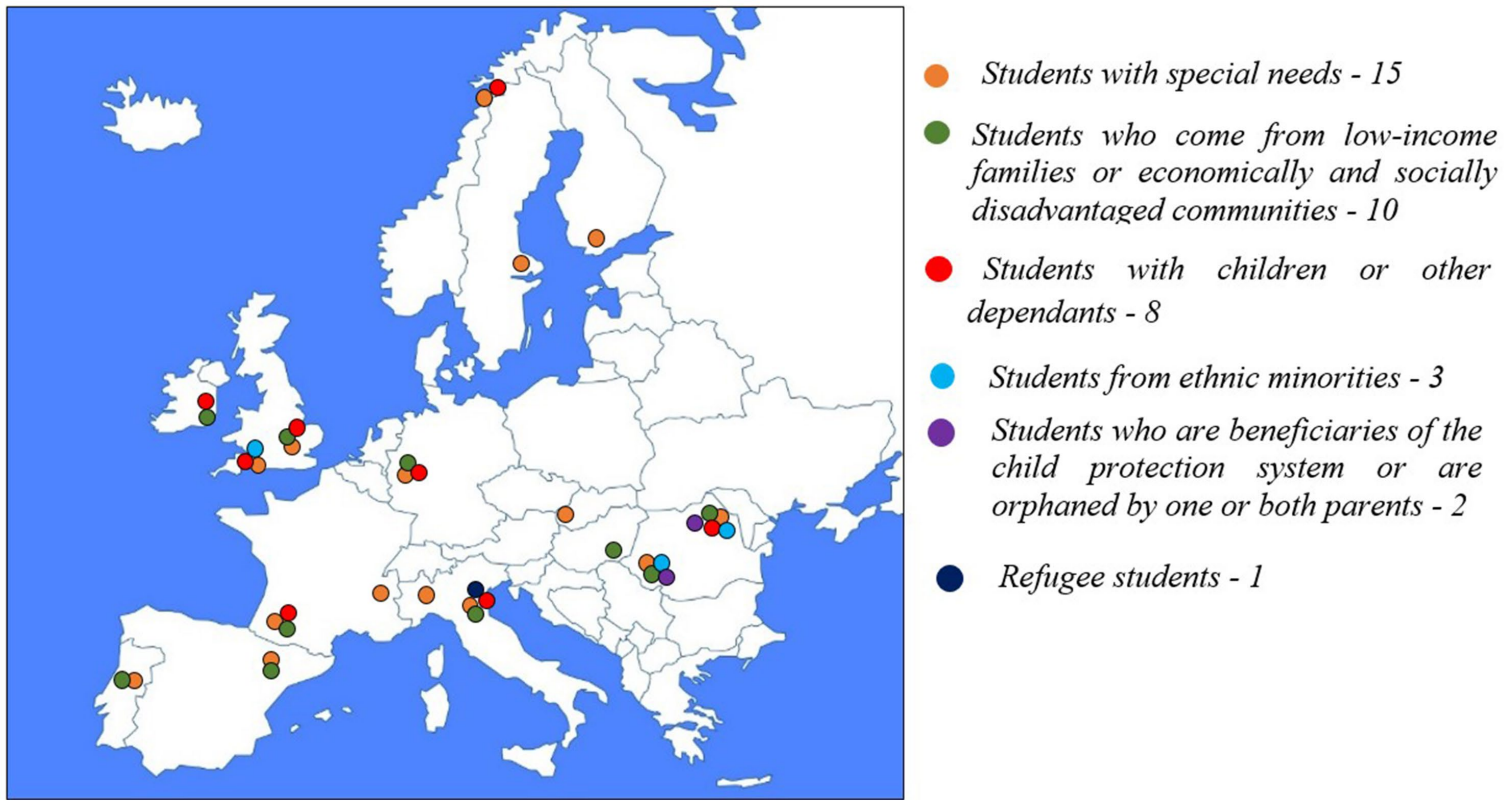
Population categories targeted by the universities in the design of support measures (both types of measures).

Six different categories of students were identified as target groups for the support measures provided by the universities (see Figure 2; Table 3):

1. *Students with special needs* are frequently the focus of universities’ specialized support measures: 15 universities out of a total of 17 in the sample provided information about the support measures made available to them. To these students, the universities typically provided a combination of financial aid measures, and support and adaptation services.
2. *Students who come from low-income families or economically and socially disadvantaged communities* were the focus of support measures in 10 of the 17 universities examined. It should be noted that there are 3 types of eligibility criteria for this category: (1) criteria related to family income; (2) criteria related to the specific economic and social condition of the students’ community of residence (e.g., certain deprived neighborhoods in the urban space in U.K.); and (3) criteria that relate generically to the type of community of residence (for example, rural community, with no other predefined characteristics). The support measures provided by the universities for this category of students are mostly financial aid measures.
3. *Students with children or other dependents*. At eight of the seventeen universities, we identified support measures for students with children or other dependents in their care. While some universities (in France, Germany and Italy) provide a mix of support measures, others focus either solely on financial aid measures (in U.K. and Ireland), or only on support and adaptation services (in Norway and Romania).
4. *Students from ethnic minorities*. This category was encountered only in universities form U.K. and Romania.
5. *Students who are beneficiaries of the child protection system or are orphaned by one or both parents*—a category targeted only by the Romanian universities included in the analysis.
6. *Refugee students* were identified under this label only at one of the Italian universities in the sample.

**Table 3:** The targeted support measures, by student category and type.

Population category	Financial aid measures	Support and adaptation services
*Students with special needs* (includes: people with temporary or permanent disabilities, people with health problems and people with learning difficulties)	Scholarships, grants, allowances, reserved seats upon admission, various tax exemption systems (registration, tuition), financial support or subsidizing the purchase of materials or equipment intended to assist in the learning process, free or discounted accommodation	Adaptation/accessibility of learning spaces (from universities and libraries, including provision of parking spaces); Adapting the manner of teaching and examination (including by reducing the frequency of attendance, changing the examination method, extending the examination time, extending the schooling period, providing an assistant to take notes or write the answers during the examination); Provision of assistive technology, including advice on its use; tutoring and meditation services; Adaptation/accessibility of accommodation spaces; Academic and professional counseling and guidance; Mediating relationships with teachers; Ensuring teacher training; Adaptation of admission tests
*Students who come from low-income families or economically and socially disadvantaged communities* (including from rural areas)	Subsidized places, other types of subsidies, social grants, exemption from paying some fees	Program to prepare pupils 12 to 18 to access higher education; Employment opportunities within university structures; Free private lessons to prepare the pupils for the Baccalaureate exam (at the end of high school); Activities to identify and prevent the risk of university dropout among first-year students
*Students with children or other dependents*	Various forms of financial aid (support funds, grants) generally aimed at helping the parent to meet the costs of having the child supervised by another person (when the parent attends classes) and reduction of tuition fees; social grants	Priority accommodation or accommodation in certain types of structures that allow better fulfillment of family needs (e.g., apartments); Provision of kindergarten or nursery services, including emergency child care; Psychological support to respond to children’s needs; Partial or total exemption of obligations to attend; adapted examination sessions; Designated spaces in the university buildings for breastfeeding or changing the child’s diapers; Maternity leave for pregnant students; Counseling and psychological support services for those who have dependents other than children
*Students from ethnic minorities*	Subsidized places specifically dedicated to Roma students	Working groups and training modules mainly for staff, to combat discrimination
*Students who are beneficiaries of the child protection system or are orphaned by one or both parents*	Subsidized places, social grants, eligibility for subsidized accommodation, exemptions or reductions in certain fees	
*Refugee students*	Financial support through grants	Support for cultural integration, “University corridors” for refugees (aimed at supporting young refugees in achieving an academic path in Italy)
*All students (no vulnerability criteria required apriori)*	Scholarships whose awarding is contingent on attaining a minimum level of academic achievement; Discounts or subsidies for public transportation and food (in university canteens)	Promoting an inclusive, participatory and non-discriminatory climate for all students, by implementing policies (gender equality), services (student service, psychological counseling services), training programs (for teachers and students) and specific support (student advocate); Promoting the well-being of students, by providing services (emergency service on campus), spaces (meditation space) and actions (referral to specialized services in the community, religious assistance) aimed at maintaining their well-being; Special offices/services or welfare officers for students—aimed at assisting students when in need for support or representation; Counseling and guidance services, including career guidance; Psychological support and guidance services; Tutoring services; Plans and strategies for gender equality and/or anti-discrimination, with specific offices for their implementation; Ombudsman/ Ombudsperson or Student Advocate—a person or an office to whom students can turn with complaints, notifications, etc. and which mediates their resolution.

**Figure 3:**
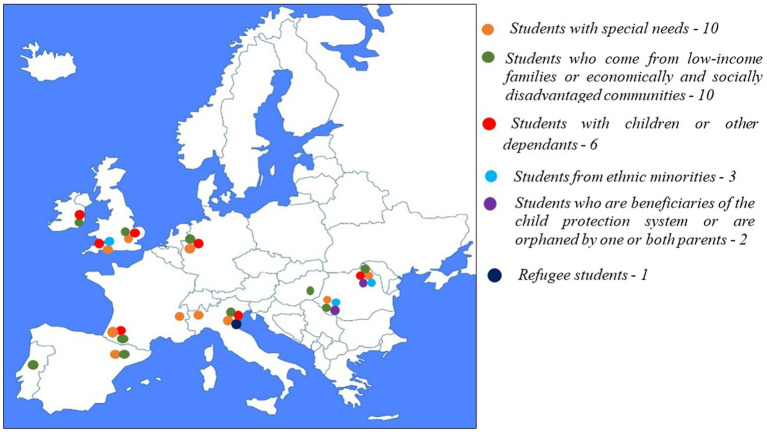
Population categories targeted by the universities in the provision of financial aid.

**Figure 4:**
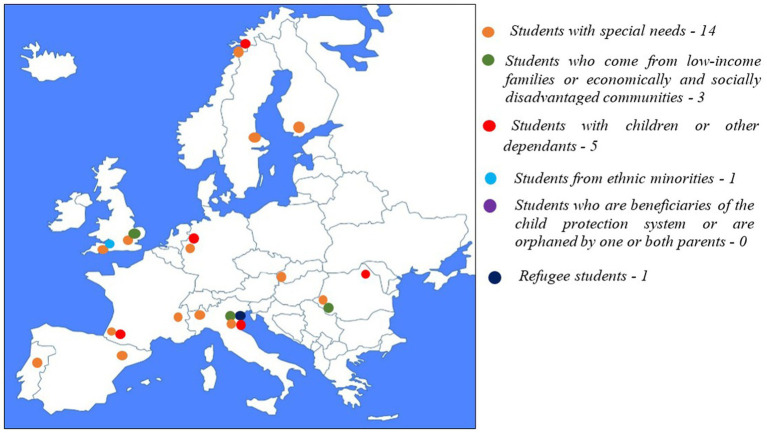
Population categories targeted by the universities in the provision of support and adaptation services.

The breakdown of the support measures by student categories and types of support is presented in Table 3, Figure 3 (financial aid), and Figure 4 (support and adaptation services).

### 3.2 Support measures provided to students

To students who comply with certain social vulnerability criteria, *the financial aid measures* provided by the universities include: discounts, subsidies, or free access to housing, public transportation, canteens, and health services, scholarships, grants, subsidies, allowances, and tax exemptions (for registration, tuition etc.).

In addition to these, universities also provide scholarships, designed to stimulate student performance, whose awarding is contingent on attaining a minimum level of academic achievement (obtaining a minimum grade, accumulating a minimum number of study credits, etc.), but without being correlated with the fulfillment of certain criteria of social vulnerability.

Two of the seventeen universities used criteria related to both the vulnerability background and the academic performance when selecting the student beneficiaries of certain types of financial aid: one of the Italian universities, where high-performing students from low-income families can get jobs in university structures, and the German university, which pays for research assistantships for first-generation students.

The support and adaptation measures are meant to cover a large variety of student needs and range from adaptation of infrastructure, priority to accommodation facilities, counseling and welfare services, mental health services, induction programs, tutorship, adaptations of academic schedule, teaching process, examination, assistive technologies, career guidance, labor market integration services, employment opportunities within the university, kindergarten and nursery services to assistance in formulating complaints, emergency help-line, and assistance for victims of harassment and abuse.

Among the 17 universities from the sample, eight (the three universities from the Nordic countries, one university in U.K., one in Ireland, one in Germany, one in Spain, and one in Italy) have created the position of Student Ombudsman or Ombudsperson (or Students’ Advocate) within their institutional framework, collaborate with the national Ombudsman or fill a comparable position under a different title. The person in this position is in charge of investigating and handling student complaints about their interactions with the educational institution. The Ombudsman’s primary goal is to find and implement solutions to the reported issues through mediation and negotiation.

When it comes to vulnerable student categories *targeted* by these services, we observed that the universities in the sample provided the largest variety of support and adaptation services for the students with special needs.

In addition to measures aimed at certain vulnerable categories of students, the majority of the universities included in the analysis have found ways to *mainstream* some support measures, by addressing them to all students, not just to those categorized as vulnerable. The universities typically use a combination of these strategies (targeting vs. mainstreaming), though the relative proportions of each differ between them. In some cases, these support measures are also provided through student organizations.

**Figure 5:**
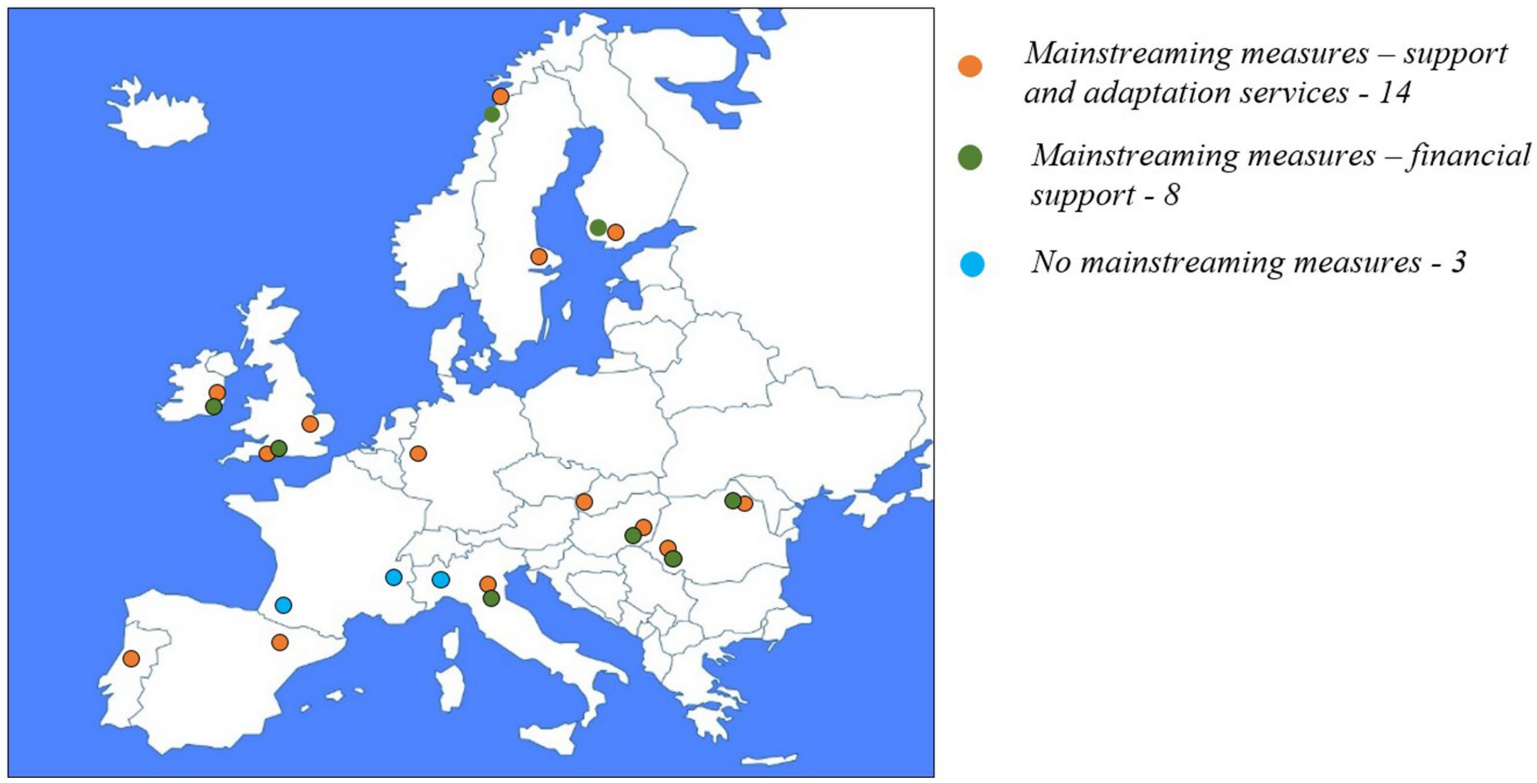
Distribution of mainstreaming measures by type of measure (financial aid vs. support and adaptation).

When it comes to the mainstreaming strategies, support and adaptation services are more popular than financial aid. Thus, 14 of the sampled universities provide at least some form of support and adaptation measures to the entire student population. Among them, 8 also provide some form of financial aid to all students (without conditionalities regarding vulnerable backgrounds). The universities that provide exclusively mainstreaming measures that rely on support and adaptation services are situated in: Sweden, U.K., Portugal, Spain, Germany, and Slovakia. No university from those included in the sample provides exclusively mainstreaming measures that rely on financial aid. The universities that provide both support and adaptation measures and financial aid to the entire student population are situated in: Norway, Finland, U.K., Ireland, Italy, Hungary, and Romania. Three of the universities in the sample have no mainstreaming measures. Two of them are situated in France and one in Italy. The breakdown of the mainstreaming measures by type of support (support and adaptation services vs. financial aid) is presented in Figure 5.

### 3.3 Welfare regimes “patterns”

The three universities from the Nordic countries (associated with the social-democratic welfare regime) lack measures of targeted financial aid, while the targeted support and adaptation is directed mainly towards students with special needs. Additionally, they use a very limited number of mainstreaming measures, both in terms of financial aid and support and adaptation.

The three universities from U.K. and Ireland (associated with the liberal welfare regime) use a mix of measures (financial aid and support and adaptation), directed both to specific target groups (students with special needs, students from vulnerable families or disadvantaged communities, students with children and other dependents, students from ethnic minorities), and to the entire student population.

The three universities in France and Germany (associated with the continental or conservative regime) lack mainstreaming measures (both in terms of financial aid and support and adaptation) and rely completely on targeted measures. The preferred categories for both types of measures (financial aid and support and adaptation) are: students with special needs, and students with children.

Among the four universities in Italy, Spain and Portugal (associated with the Mediterranean regime) we also observe a significant preference for targeted measures. The categories targeted by financial aid measures are: students with special needs, students from low-income families, students from rural areas, refugee students, and students with children. The most frequently mentioned category targeted by the support and adaptation measures is by far that of students with special needs.

Among the four universities in Hungary, Slovakia and Romania (associated with the post-socialist regime) we observe very different patterns: the Hungarian university uses financial aid as a preferred measure, directed to various categories of students. But most of these categories do not fall under the “vulnerability” umbrella, as we described it at the beginning of this paper. On the other hand, the Slovak university does not use financial aid, but relies only on the support and adaptation measures, addressed to all students and to students with special needs. The two Romanian universities also rely mostly on financial aid, that is addressed both to all students and to students from vulnerable categories. The categories that are targeted by these measures are the most diverse in the entire sample: students who are orphaned or are beneficiaries of the child protection system, students from rural areas, students from ethnic minorities, students with special needs, students with children, students with chronic illnesses, students from low-income families.

Thus, when it comes to the balance between mainstreaming and targeting strategies, we observe a preference for targeted measures in universities from countries conservatively associated with social-democratic, continental/conservative, and Mediterranean welfare regimes. While in universities from the two countries conservatively associated with the liberal welfare regime we observe a mix of mainstreaming and targeted strategies, in the universities from countries associated with the post-socialist regime there is no discernable preference.

However, when it comes to the balance between financial aid and support and adaptation measures, we observe that, while the universities from countries conservatively associated with the social-democratic welfare regime use very little or no financial aid measures, all the other universities use some sort of financial aid measures, with the distinction that in the countries conservatively associated with continental/conservative and Mediterranean welfare regimes this financial aid is mostly targeted to specific categories. The most varied support and adaptation measures addressed to the entire student population are found in the universities for the two countries conservatively associated with the liberal welfare regime.

## 4 Discussion

The support measures aimed at enhancing the access of students from vulnerable groups to tertiary education vary among the universities examined, and the differences can be attributed to a range of factors operating at different levels of analysis (macro, mezzo, and micro), including: national policies, legislation, and regulations; the specific type of welfare regime prevalent in the country; the cultural values that shape societal norms and expectations regarding the university’s role; the social and demographic characteristics of the vulnerable population in the targeted region; the resources (encompassing financial, physical, material, and human assets) available to the university; as well as the number and profile of the students being served. This is in line with the observations of other researchers about the differentiation of European universities, explained through their internalization of larger-scale, national policy arrangements meant to ensure the social equity (Brennan and Naidoo, 2008; Hüther and Krücken, 2016). However, beyond these distinctions, numerous similarities can be observed.

In the following, we will discuss the support measures undertaken by the sampled universities considering a Foucauldian perspective, which pertains to the connection between education and governance. Foucault (1982, 2008) emphasizes the need for examining the interconnectedness of various power techniques and the emergence of broader forms of power. He directs our attention towards understanding how these techniques and dimensions of power are networked (Hannus and Simola, 2010).

### 4.1 Targeted measures and mainstreaming strategies

As presented in the previous sections, most support measures provided by the universities are *targeted* to specific categories of students, defined as vulnerable (namely students with special needs, students who come from low-income families or economically and socially disadvantaged communities, students with children or other dependents, students from ethnic minorities, students who are beneficiaries of the child protection system or are orphaned by one or both parents, refugee students). Therefore, any student who wants to access these support measures has to prove they fit with the pre-defined vulnerability criteria that place them into a category or another, thus becoming a willingly subject of the power exercised by the universities through the formulation of their policies and regulations.

Foucault’s concept of power extends beyond overt control to include the subtle, pervasive ways in which institutions shape and influence individuals. In this context, targeted support measures can be seen as mechanisms for categorizing and managing students based on pre-defined vulnerabilities. Such categorization serves as a form of surveillance, with students monitored and evaluated based on their compliance with institutional expectations, defined through eligibility criteria.

The *eligibility criteria* employed to identify students for targeted interventions implicitly communicate specific ideas, values, and concepts that are typically associated with the ruling collectives (Calderón-Almendros and Ruiz-Román, 2016). These normative criteria are intended to legitimize and advance specific identities and collectives, as well as to persuade and standardize others (Foucault, 2002). All of this is the result of intricate and subtle processes of legitimation and appropriation of individuals and groups. In this manner, an exchange occurs whether to include individuals within a specific collective or not through the legitimating identity. The legitimating identity encourages individuals to internalize and embrace the meanings of the cultural community as the most viable option, as it best ensures their integration and development within the community (Calderón-Almendros and Ruiz-Román, 2016), as well as their accessing the specific support measures.

By labeling certain students as “vulnerable”, universities may impose (even if unintentionally) disciplinary power, resulting in the internalization of these identities by the students, and the creation of the “docile bodies”, in which individuals are trained to follow institutional norms and practices (Foucault, 1995).

At the same time, the implementation of targeted inclusion policies in universities may result in processes of othering of those who are targeted by such policies (Dunne, 2009). For example, Bacchi (1996) analysis of the effects affirmative actions, as a base for social policies, produce on their target groups in terms of social change, differ depending on their interpretations. When viewed as “special treatment”, they resulted in restricted reform to ameliorative measures targeted towards “disadvantaged” groups, to assist them to compete and integrate. On the other hand, when viewed as a means of achieving social justice, they created the space and context for significant revisions of hiring and promotion policies.

Thus, targeted measures of support can reinforce existing power structures by emphasizing deficiency and risk, this approach limiting students’ agency by assigning them to predefined roles that align with the institution’s perception of vulnerability. Universities use such services as *biopower tools* to regulate and optimize the activity and identity of their students by managing those who fit the criteria of vulnerability (Foucault et al., 2008). In this sense, targeted measures are part of a larger strategy of governmentality, in which the discourse employed through university policies is utilized to define categories, label identities and set expectations for students to comply with.

However, the targeted support measures are not the only support measures employed by the universities in the sample. As mentioned in Section 3.2 and in Table 2, most universities in the sample also provide support measures for the entire student body, using a *mainstreaming* strategy of support.

The design of these support measures makes them more accessible, because they do not define very strictly their target population and, thus, are not provided based on apriori means-testing processes. Thus, the students who request them do not feel the pressure to prove they fulfill the eligibility criteria before addressing for help. This way, any student who needs help at a certain point, will feel free to ask for it.

However, mainstreaming strategies are not free of power dynamics and, from a Foucauldian standpoint, they can be interpreted as encouraging *normalization*. This normalization process can obscure vulnerable students’ unique needs by focusing on creating a homogeneous student body rather than addressing individual differences. The influence of normalization is facilitated by its ability to appear impartial, which is achieved through the use of specialist jargon or the reference to the “neutral” truth, facts, and common sense (Kopecký, 2011). The mainstreaming strategy can also be viewed as a form of governmentality which accentuates personal responsibility and positions students as self-governing individuals responsible for their own success and well-being (Ball, 2012).

Regarding the preference for one of the two strategies, it is worth noting that imagining a system that operates cohesively and which achieves its goals solely through either strategy is difficult. Although some scholars argue for targeted rather than mainstreaming strategies to ensure equity in higher education (Salmi and D'Addio, 2021; Salmi and Malee Bassett, 2014), it is critical to also recognize the specific benefits of the mainstreaming strategies: (1) they provide a wider safety net, offering solutions for a broader range of needs, including those that have yet to be detected or defined and (2) they prevent the fragmentation of support services that is inherent in the targeting strategy.

### 4.2 Monetary and non-monetary support

Most of the universities from the sample employ a combination of financial aid and support and adaptation services for vulnerable students to promote an equitable environment. Financial aid is frequently given to students from low-income families, economically disadvantaged communities, and other specific groups, such as those with dependents or those in the child welfare system. This financial assistance includes scholarships, grants, and subsidies for tuition, housing, and transportation, with the goal of removing immediate economic barriers to accessing higher education (Salmi and D'Addio, 2021; Santiago et al., 2008; Salmi and Malee Bassett, 2014). From a Foucauldian perspective, financial aid can be viewed as a biopolitical tool used for positive aims, such as reducing economic stressors and improving students’ wellbeing. Indeed, the financial resources provided by the universities may act as motivating mechanisms, which shape students’ effort and willingness in attaining academic performance (Foucault et al., 2008).

At the same time, they reinforce the position of power the university has in relationship with the students, especially when granted based on previously proven academic performance measured through grades. Grades obtained as a result of testing integrate the methodologies of hierarchy and sanctions. They enhance the authority of the norm and accentuates individual responsibility and homogeneity. Based on Foucault’s analysis of technologies that uphold normalizing power, scholars have shown that various student assessment techniques directed to shape specific model citizens can generate normalizing and othering effects (Simola, 2002).

Hellbert-Steurer (2018) points out that, while in the welfare state, the individual is perceived as a social citizen, interconnected with others within a shared community, in a neoliberal government, the individual is characterized as an autonomous, calculative agent pursuing self-interest and competing with others in a free market economy. The neoliberal governmentality is emphasizing freedom and autonomy, by instilling a sense of self-determination and independence, but, at the same time, governs human behavior by methods of surveillance, performance assessment, and evaluation, rendering the individual reliant on societal acknowledgment of their actions.

Thus, financial monetary support adheres to neoliberal principles by emphasizing individual responsibility for managing one’s financial resources (Brown, 2015), reinforcing the narrative that financial management is a personal responsibility, promoting self-governance in accordance with broader neoliberal ideologies. As a result, this emphasis on individual responsibility may limit the university’s ability to address more complex social and structural issues affecting students’ economic circumstances.

However, financial aid alone is frequently insufficient to meet the broader and often more complex needs of vulnerable students. Students with disabilities, for example, or those who have children in their care, require specialized support services, such as academic accommodations and adjustments. These needs go beyond financial assistance and necessitate a more holistic approach to support and adaptation (Usher and Burroughs, 2018). In the opinion of other authors, the direct support services such as counseling, tutoring, and mentoring represent another form of institutional intervention that shapes subjectivity, by normalizing certain behaviours and attitudes in alignment with institutional expectations (Foucault, 1995; Gore, 1995). Counseling services, for example, produce a process of subjectification, by shaping students’ compliance with institutional norms regarding mental health and academic performance, resulting in a more overt form of power in which institutions have direct influence over students’ identities, capabilities and actions (Nicoll and Fejes, 2008).

The universities’ governance through the provision of financial aid, encourages students to manage their financial well-being in accordance with institutional goals, thereby increasing the university’s influence over students’ lives without direct intervention. On the other hand, direct support services involve a more active and multi-dimensional form of governance, addressing students’ academic, social, and emotional components and shaping more closely their educational experiences (Rose, 1999). Ball (2016) asserts that one of the ways in which neoliberalism has profoundly transformed the nature and purpose of education is by shaping specific approaches to conceptualizing and managing one’s academic identity through ethical and discursive dimensions (Sojot, 2018).

Balancing between financial aid and direct support services represents a complex interplay of power dynamics within educational institutions: financial aid is consistent with neoliberal governance and emphasizes individual responsibility and self-regulation, whereas direct support services require more direct forms of surveillance and control. Each approach has different implications for how universities govern and influence vulnerable students’ experiences, defining their agency and subjectivity within the educational context.

### 4.3 Student agency exercised through student Ombudsman and student organizations

Contemporary scholars have expanded on Foucault’s theories to investigate how institutions such as universities use governance mechanisms to manage student populations. For example, Brown (2015) discusses how institutions frequently frame student services, including Ombudsman roles, within a neoliberal framework that values autonomy, individual responsibility and self-management. However, Peters et al. (2000) point out that, in neoliberalism, individuals cannot be perceived as free, as the frameworks of reason and the regulations governing the free market will mold them into specific types of subjects, thereby influencing their choices in predictable manners.

From a Foucauldian perspective, the Ombudsman’s role within the university structure can be seen as another mode of governing, in which power, governmentality, monitoring, and normalization intertwine in subtle ways. On the one hand, by providing students with a formal way to express their concerns, the Student Ombudsman ostensibly empowers them by giving them some say in the university system. Thus, the Ombudsman role creates a form of counter-power within the institution by providing a forum for mediation and negotiation, allowing students to challenge decisions or practices that they believe are unfair. On the other hand, this empowerment also serves as a tool for institutional *control and regulation*, as its power is set within the boundaries of the system. As part of the structures of power within higher education institutions, although appearing to advocate for students, the Ombudsman follows university policies and ultimately serves to maintain institutional order and stability. By channelling dissent into controlled forms, the role reduces the likelihood of more disruptive challenges to the institution’s authority. This is consistent with Foucault’s view that institutions exercise power not only through direct coercion but also through mechanisms that encourage self-regulation and conformity (Lemke, 2015). Under this framework, the Ombudsman’s role comes in line with Foucault’s *pastoral power theory* (Foucault, 2007), which asserts that institutions serve as guides under the guise of benevolence: while responding to student complaints, the Ombudsman gently guides students towards appropriate behaviours and interactions with the institution.

Additionally, the Ombudsman serves a process of *normalization* that reinforces existing power dynamics and institutional norms (Foucault, 2003): it teaches students to solve conflicts through institutionalized channels.

Furthermore, the Ombudsman’s role is part of a larger university surveillance system. It provides a forum for students to express their concerns while also allowing the institution to *monitor* and address them. This is a type of disciplinary authority in which the institution monitors and regulates individuals through subtle, pervasive forms of observation (Garland, 2014). The Ombudsman’s documentation and resolution of student grievances help facilitate this process by allowing the institution to maintain control over the student body while appearing responsive to their needs.

The downside of this approach is that it limits the university’s engagement with larger structural inequalities by emphasizing immediate issues rather than addressing underlying systemic ones.

However, there are two other mechanisms through which student can exercise their agency in universities: (1) student organizations and (2) student representation in university governing bodies. The student organizations, close partners to many of the universities in the sample, are, at least in theory, the structures that embody students’ agency. In an essay about student organizations’ protests in Italy, Schiavo (2018) addresses the importance of the power exercised through student mobilization and protests, in opposing austerity measures in the Italian universities, dictated under neoliberal organization principles.

Additionally, Toliou (2007) underlines that the requirement of student representation in university governing bodies is a useful tool for balancing the power distribution in the universities and ensure the students interests are well served.

## 5 Concluding remarks

In this study, we have sought to understand (1) how European universities problematize inequality in higher education, by labeling and categorizing vulnerable students and (2) what modes of governing (designed as support measures) they employ in addressing inequality. Using a Foucauldian lens to analyze the rationales and modes of governing behind the tools through which public European universities put their social dimension into practice, allows us to take them outside the logic of the discourse within which they were designed and challenge (or problematize) the framing of the issue itself.

The findings of this study show that the effort dedicated by the universities in the sample to enhance access of vulnerable students to higher education, as well as the categories of students to whom the support measures are dedicated to vary from case to case.

The analysis of the relationship between financial aid and direct support services employed by the universities exemplifies a complex interplay of power and normalization: financial aid, as framed by neoliberal principles, emphasizes personal responsibility and self-regulation (Brown, 2015), whereas direct support services, as viewed through Foucauldian lenses, serve as tools of surveillance and normalization, shaping student behavior to align with institutional norms (Foucault, 1995; Gore, 1995).

In a similar vein, the universities’ oscillation between targeted and mainstreaming strategies reflects different types of governance and institutional control: targeted services categorize, label and manage students based on perceived vulnerabilities, which can reinforce power dynamics and limit agency (Foucault, 1995); mainstreaming strategies, on the other hand, aim for inclusivity but may impose standardized norms that marginalize those who do not conform (Foucault, 1995).

The Student Ombudsman’s, although a measure apparently supportive of student agency, in addition to providing a platform for student grievances, is also useful in exercising institutional control, by channelling dissent in a way that maintains order and reinforces institutional norms (Foucault, 1995; Lemke, 2015). In exchange, student organizations appear as more straightforward promoters of student agency, through grassroots mobilization (Schiavo, 2018). Previous studies on disadvantaged students’ agency in the context of higher education (Smith, 2007; Danic, 2015) show that the enrolment itself in higher education programs is a way they exercise this agency to overcome the lower social status assigned to them by wider structural and institutional arrangements that work at societal level.

The analysis of each approach revealed the complexities of how universities, acting within the larger frameworks of social and educational policies, promote equality by providing support to vulnerable students. It also revealed how each approach generate different power dynamics between these two actors. In these power dynamics at local scale we see at work the bio-power tools employed by the universities to govern students’ lives, by shaping their identities and behaviours, in accordance with pre-established ideals of functionality, integration, and performance, as defined by the policies under which they function.

The analysis also allows us to notice two additional aspects: (1) a preference of universities to engage with smaller-scale issues than with systemic ones, as well as (2) a widespread use of measures which accentuate responsabilization of the individual.

Integrating the findings of the study into a larger discussion of modes of governing in higher education reveals that the mechanisms used by universities to support vulnerable students are inextricably linked to larger institutional and societal dynamics.

Foucault (1980) argues that rule occurs by means of subjects, or more precisely, through the creation of governable subjects. The process of subjectification refers to the way in which individuals are constructed as subjects. Assuming specific “subject positions” involves embracing specific self-perceptions and embodying a particular identity. It implies that policies have the power to shape our subjectivities by creating specific subject positions, and that power relations shape our self-perception (Bacchi, 2010). Bonal (2003) describes how, under neoliberal political rationality, in spite of the fact that individual and collective behavior exhibit formal freedom, their behavior is actually shaped by the governance structures. Thus, the person gradually transitions from being subject of rights to being subject of duties, by needing to prove their entitlement to rights and entitlements. By claiming to articulate the reality of how societal, organizational, and individual structures should work, neoliberal discourse becomes inescapable and imposes a normative nature to its truths, which legitimizes economic actions inside the educational sector and allows the uneven power dynamics to perpetuate (Hellbert-Steurer, 2018).

Pasias and Roussakis assert that the methodologies employed to achieve a Europe of Knowledge appear to have been `colonized` by the neoliberal and technocratic principles of marketization, privatization, governmentality, and performativity, as advocated by international economic entities, as well as transnational corporations, which subtly shape educational discourse (Pasias and Roussakis, 2012: p. 183).

Clearly, the universities’ engagement with their social dimension involves devoting significant resources (mostly financial, but also time and specialized personnel) to this social commitment. Biesta (2015) observes that normative inquiries regarding (good) education have been supplanted by technical-managerial perspectives that emphasize processes over purposes in education. Over the last twenty years, the normative concept of education has diminished while, at the same time, discussions surrounding measurement, quality, and qualification have increased.

Ball (2016) notes that the emphasis on technologies of performance within neoliberal frameworks is apparent in various quality assurance mechanisms, standards, measurements, and outputs, all oriented towards productivity and growth, often described as “governing by numbers”. The neoliberal focus on performance in education, has resulted in the degradation of care.

Besides the high financial and operational costs of supporting vulnerable students, there is also the matter of the higher dropout risk among the vulnerable categories of students. Dropout rates have an impact not only on institutions’ quality benchmarks, but also on their ability to secure future funding (Guri-Rosenblit et al., 2007), and, as a result, universities may be less motivated to allocate resources to students with lower academic performance, as these investments may not always align with their strategic goals or funding criteria (Archer, 2007).

When the governance of higher education aims at efficient resource management, the management of metrics establishes the parameters of how knowledge is defined and should function. These parameters, in turn, constrain the research collaborations between universities and communities (Gibbons, 2018).

As Calhoun (2006) noticed almost two decades ago, some of the characteristics of higher education, as they were designed at that time and still remained so now, hamper the attainment of ambitions related to equality, equity and accessibility. The current quality assessment criteria used in higher education, which primarily focus on performance and excellence are not sufficiently calibrated with social goals such as that of promoting diversity.

Therefore, the European universities are required to handle an inherent tension between the social welfare objectives they are expected to achieve (equality, equity, inclusivity) and the neoliberal climate that describes their current context (characterized by financial constraints, competition, harsh assessment criteria).

To address these challenges and improve the engagement of European universities with the social dimension of higher education, scholars advocate for two key policy actions: (1) incorporating the social dimension into the quality criteria used to evaluate universities (Nyssen, 2018; Vlk and Stiburek, 2018; United Nations Educational, Scientific and Cultural Organization, 2009) and (2) giving universities more autonomy in setting their own agendas and tailoring their approaches to specific and regionalized student needs, such as adjusted admissions criteria, flexible learning paths, and improved support services (Vlk and Stiburek, 2018; Salmi, 2018a; Haj et al., 2018).

The main limitations of the analysis relate to the relatively modest volume of the sample and the availability of relevant information on the website of the universities included in the analysis. Also, another aspect that can be considered a limitation consist in the loss of local nuance in the process of gathering, structuring, unifying and categorizing data sampled from various geographical spaces, with very different cultures. In the process of translation and analysis, some phrases, ideas and expressions were simplified and thus probably lost some nuances of their original meaning.

## Data Availability

The datasets presented in this article are available upon reasonable request. Requests to access the datasets should be directed to theofild.lazar@e-uvt.ro.
